# Epidermal growth factor receptor and myeloperoxidase expression in geographic tongue: an observational study

**DOI:** 10.3389/fimmu.2026.1924136

**Published:** 2026-08-19

**Authors:** Amal Dafar, Anna-Karin Östberg, Asal Shikhan, Lena Larsson, Mats Jontell, Hülya Cevik-Aras

**Affiliations:** 1 Department of Oral Medicine and Pathology, Institute of Odontology, The Sahlgrenska Academy, University of Gothenburg, Gothenburg, Sweden; 2 Department of Oral and Maxillofacial Surgery, King Fahad General Hospital, Jeddah Second Health Cluster, Ministry of Health, Jeddah, Saudi Arabia; 3 Department of Oral Microbiology and Immunology, Institute of Odontology, Sahlgrenska Academy, University of Gothenburg, Gothenburg, Sweden; 4 Department of Periodontology, Institute of Odontology, Sahlgrenska Academy, University of Gothenburg, Gothenburg, Sweden; 5 Section for Oral Biology and Immunopathology/Oral Medicine, Department of Odontology, Faculty of Health and Medical Sciences, University of Copenhagen, Copenhagen, Denmark

**Keywords:** benign migratory glossitis, growth factors, myeloperoxidase, oral diseases, oral mucosal lesions, tongue lesions

## Abstract

**Background:**

Geographic tongue (GT) is a common oral mucosal lesion that affects the dorsal and lateral surfaces of the tongue and characterized by erythematous areas of papillary atrophy surrounded by peripheral zones. The aim of this study was to evaluate the expression of myeloperoxidase (MPO), epidermal growth factor (EGF) and epidermal growth factor receptor (EGFR) in tissue samples from patients with GT compared to control.

**Methods:**

Biopsies specimens from patients with GT (n=10, mean age 48.3 ± 16.7 years) and control subjects (n=3, mean age 59.0 ± 3 years) were evaluated. Immunofluorescence was used to assess the MPO staining intensity of positive cells (i.e. scoring grades 0, 1 and 2). Furthermore, the percentage of EFG and EGFR positive cells in the tongue tissue samples was evaluated by immunofluorescence.

**Results:**

Most of the patients with GT (n=6) scored 2 for MPO expression, demonstrating an increased expression of MPO among GT patients compared to controls (*p* = 0.0266). The expression of EGF positive cells among patients with GT showed no difference compared to controls. In contrast, patients with GT showed a statistically significant low EGFR activity compared to controls (*p* < 0.0001).

**Conclusion:**

Patients with GT showed a high MPO activity while low EGFR expression which may explain the underlying inflammatory response and decreased regeneration process in GT.

## 1 Introduction

Geographic tongue (GT) is a common inflammatory lesion of the oral mucosa affecting the dorsal and lateral surfaces of the tongue. A recent systematic review and meta-analysis estimated its worldwide prevalence among adults to be approximately 3% (1). Clinically, GT is characterized by well-demarcated erythematous areas representing papillary atrophy, surrounded by slightly elevated whitish or erythematous borders. A hallmark of the condition is the continuous migration of these lesions, resulting in the characteristic map-like appearance (2). Although GT is often asymptomatic, some patients experience soreness, burning sensations, or increased sensitivity of the tongue, particularly after consuming spicy foods or acidic beverages (3).

The exact aetiology and pathogenesis of GT remain unclear. However, the condition is generally regarded as a reaction pattern of the tongue in which multiple factors, including inflammation and altered tissue regeneration, may contribute to disease development. Histopathologically, GT is characterized by subepithelial inflammatory infiltrates predominantly composed of neutrophils (4). Supporting the involvement of neutrophil-mediated inflammation, salivary levels of the neutrophil chemoattractant interleukin-8 (IL-8) have been reported to be significantly higher in patients with GT than in healthy controls (5).

Myeloperoxidase (MPO) is a heme-containing enzyme expressed predominantly by neutrophils. It catalyses the formation of reactive oxygen species during the respiratory burst, thereby contributing to microbial killing while also participating in the regulation and resolution of inflammation (6). Because excessive MPO activity may also promote tissue injury, MPO has been widely investigated as a biomarker of inflammatory activity and tissue damage in a variety of inflammatory disorders (7). Its potential role in GT, however, has not yet been fully elucidated.

The mechanisms underlying the papillary atrophy characteristic of GT are also poorly understood. Under physiological conditions, the lingual epithelium undergoes continuous proliferation, differentiation, maturation, and apoptosis, processes regulated by several growth factors, including epidermal growth factor (EGF) (8), and vascular endothelial growth factor (VEGF) (9). Among these, epidermal growth factor receptor (EGFR) signalling plays a central role in epithelial cell proliferation, differentiation, survival, and tissue repair (10). Altered salivary EGF levels have previously been reported in patients with GT, suggesting a possible involvement of the EGF/EGFR signalling pathway in the pathophysiology of the disease. This hypothesis is further supported by experimental studies demonstrating that removal of the submandibular glands in rats induces lingual papillary atrophy, which can be reversed by EGF replacement (11).

Based on these observations, we hypothesized that both neutrophil-mediated inflammation and altered epithelial growth factor signalling contribute to the pathogenesis of GT. Therefore, the aim of the present study was to investigate the tissue expression of MPO, EGF, and EGFR in tongue biopsies from patients with GT compared with healthy control subjects.

## 2 Materials and methods

### 2.1 Participants

In this observational cross-sectional study, ten patients diagnosed with GT (7 males and 3 females, mean age 48.3 ± 16.7 years, range 24-65) and 3 control subjects with no oral mucosal diseases except for irritational fibromas (2 males and 1 female, mean age 59.0 ± 3 years, range 56-62) were recruited from the Clinic of Oral Medicine, Public Dental Health, Region Västra Götaland, Sweden. Incisional biopsies using a 4-mm punch were obtained from the sites of GT. For control subjects, irritational fibromas from the tongue were excised and histopathological reports for the three controls were signed out as reactive hyperplasias.

### 2.2 Fluorescence staining for MPO

The specimens were formalin-fixed, dehydrated, and then paraffin-embedded. The sections of 5 μm thickness were retrieved from -70 °C freezer, thawed at room temperature and then incubated at 60 °C for 1h. Thereafter, deparaffinization was made after which the slides were placed in a pressure cooker with citrate buffer at 110 °C for 7 min and followed by cooling to 90 °C. After that, slides were rinsed using tap water for 10 min and then using TBS for 3 min. Slides were incubated in blocking buffer of 2% donkey serum, 1% BSA, 0.1% Triton X in TBS for 30 min. Subsequently, incubation with primary antibodies for MPO polyclonal rabbit anti-human (A0398) from Dako (concentration 1:500) was done overnight (**Table 1**). The next day, slides were triple-rinsed in TBS for 5 min and then incubated with secondary antibody (Alexa Fluor 647, dilution 1:200) at room temperature for 1h. Slides were then triple-rinsed in TBS for 5 min. Finally, sections were mounted with Prolong Gold antifade reagent with DAPI (Invitrogen) and preserved in darkness until analysis. The samples were randomized and blinded preceding imaging, which was performed using the Olympus BX41 microscope (Olympus, Hamburg, Germany) equipped with the Olympus DP71 camera and the Olympus Plan C N, 10x/0.25 ∞/- FN22 objective together with the Olympus cellSens 1.13 software. Sections from tonsils tissues were used as positive control while omission of primary antibodies served as negative controls.

**Table 1:** Primary antibodies used for immunofluorescence analyses.

Primary antibody	Origin	Type	Isotype	Dilution	Company
MPO	Rabbit	Polyclonal	IgG	1:500	A0398 Dako
EGF	Mouse	Monoclonal	IgG	1:500	9D7F11 Invitrogen™ Fisher
EGFR (Phosphorylated)	Rabbit	Monoclonal	IgG1	1:100	ab40815 Abcam

### 2.3 Immunofluorescence analysis for MPO

**Figure 1:**
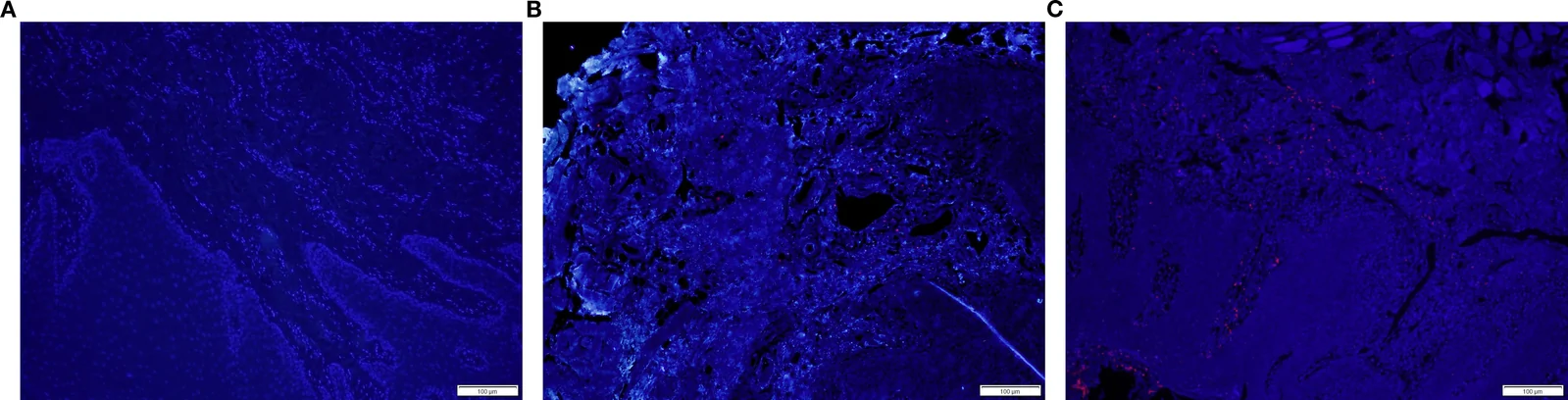
MPO fluorescence staining. Images analysed using the Olympus cellSens software showing different scores of MPO expression among patients with GT. **(A)** Score 0, negative. **(B)** Score 1, weaker than tonsils. **(C)** Score 2, equivalent to (or) stronger than tonsils. Magnification x10.

Images were analysed using the Olympus cellSens 1.13 software. The presence of MPO positive cells was evaluated and scored according to their quantity in comparison to positive controls, i.e. tonsils, as 0 = negative; 1 = weaker than tonsils; 2 = equivalent or stronger than tonsils. Two observers (AD and AKÖ) assessed the sections for scoring and disagreements were resolved by discussion. **Figure 1** illustrates different scores of MPO expression.

### 2.4 Fluorescence staining for EGF/EGFR

The specimens were formalin-fixed, dehydrated, and paraffin-embedded. Sections of 5-μm thickness were produced in a microtome and mounted on microscope slides and incubated at 60 °C for 1h prior to deparaffinization and hydration. The slides were incubated in DIVA antigen-retrieval solution (Biocare Medical, Histolab, Concord, CA, USA) at 60 °C overnight. Next day, slides were cooled down at room temperature and then washed in distilled water and in TBS for five times. For blocking endogenous activities, slides were incubated with 2% donkey serum, 4% goat serum, 4% BSA (Sigma Aldrich), and 0.1% Triton X (Sigma Aldrich) in TBS for 1h at room temperature. Thereafter, slides were incubated with primary antibodies for EGF and EGFR at 4 °C overnight (**Table 1**). Specifically, EGF Mouse anti-human (9D7F11) from Fisher (concentration 1:500) and EGFR Rabbit anti-human (40815) from Abcam (concentration 1:100) were diluted in 1% BSA (Sigma Aldrich) and 0.1% Triton X (Sigma Aldrich) in TBS. The next day, slides were triple washed in TBS for 5 min and then incubated with secondary antibodies (Alexa Fluor 488 and Alexa Fluor 568, dilution 1:100 for both) at room temperature for 2 h in the dark. After that, slides were triple washed in the dark with TBS for 5 min. DNA staining was done using Hoechst (Invitrogen, Fisher Scientific). Slides were then triple- and twice-washed with TBS and water, respectively, each for 5 min in dark. Finally, slides were mounted with ProGold. Positive cells were detected using DAB substrate (Algilent). Human oral mucosa tissues of fibroma sections were used as positive controls, while negative controls were produced by substituting the primary antibody with non-immune serum.

### 2.5 Immunofluorescence analysis for EGF/EGFR

**Figure 2:**
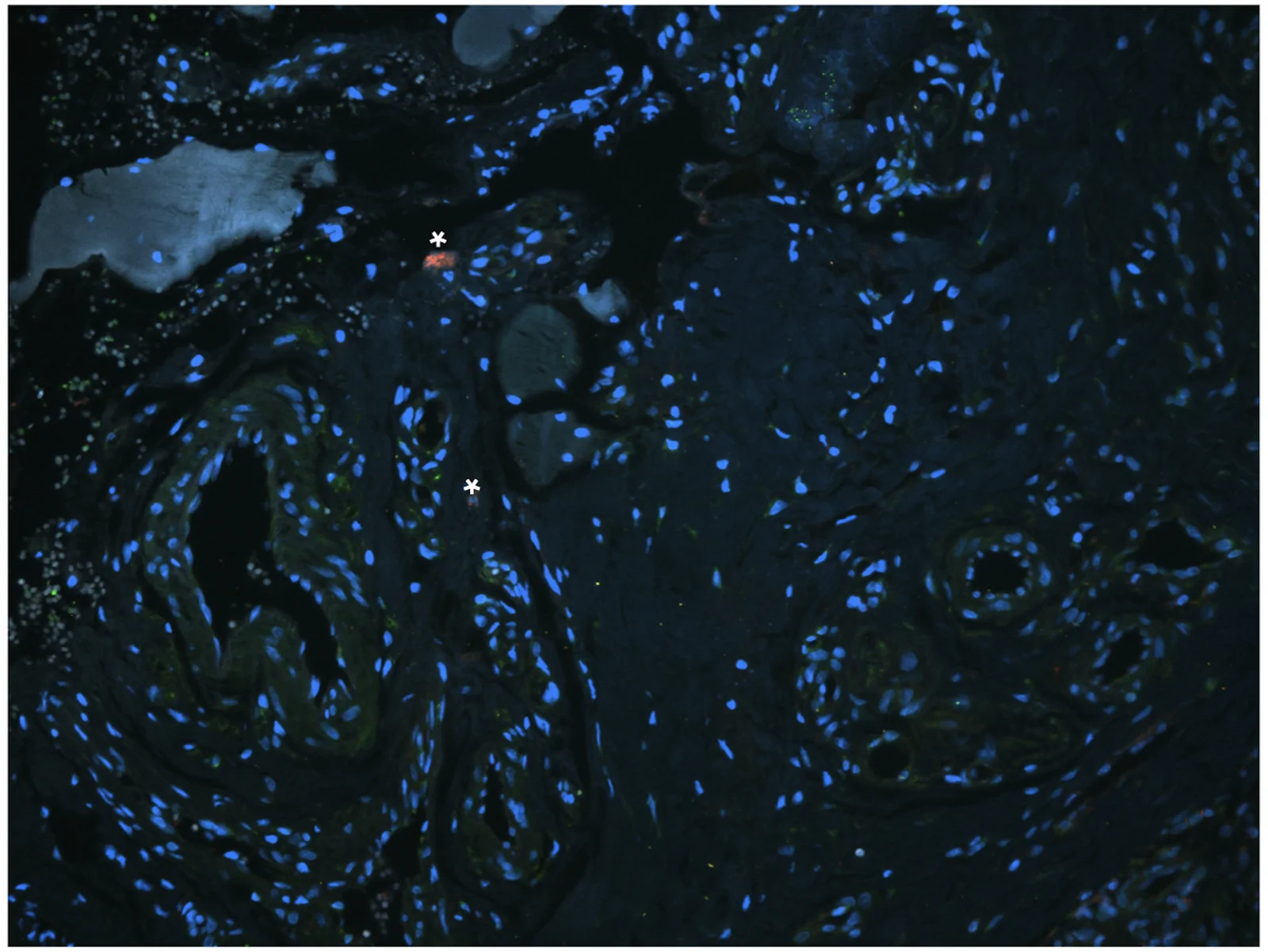
EGF and EGFR fluorescence staining. Images analysed using the software Image-Pro Premier (IPP) showing an example of EGF (green) and EGFR (red*) expression in a specimen of a patient with GT. Magnification x20.

The expression of EGF and EGFR was assessed using fluorescence microscopy of tissue sections stained with immunofluorescence. To ensure comparability between samples and controls, a consistent microscope setting throughout the analysis was maintained. The sections were systematically analysed by capturing images of the entire tissue section, and the images are saved in a separate folder to facilitate organized and reproducible data management. The images were then processed using the software Image-Pro Premier (IPP, version 9.3.3 Build 6468; Media Cybernetics Inc., Rockville, MD, USA), which is used to define and calculate the total area of the section, referred as the Region of Interest (ROI). Marker analysis, i.e. positive cells count, was performed individually by suppressing other colour channels in the software, enabling separate quantification of each marker (**Figure 2**). For each marker, the total stained area is calculated, representing the fluorescent signal generated by the specific marker within the analysed region. Finally, the measured values were used to calculate the proportion of the total section area that is stained (as a percentage) for each marker. This percentage served as the basis for further statistical analysis of the results.

### 2.6 Statistical analysis

Unpaired t-test was used to compare the age and the expression of EGF and EGFR. Fischer′s exact test was used to compare the gender. Cochran-Armitage test for trend was used to compare the MPO expression between patients with GT and controls. Statistical analyses were performed using GraphPad Prism version 10.2.3 for Mac OSX (GraphPad Software, La Jolla, CA, USA) and R Core Team (2025) version 4.5.0. The threshold for statistical significance was set at p < 0.05.

## 3 Results

### 3.1 Demographic data

There was no statistically significant difference between patients with GT and C in age and gender. Eight patients with GT were free from systemic diseases and taking no medications. The other two patients reported presence of systemic diseases. One patient reported hypertension (used amlodipine) and the other patient reported asthma, atopy and eczema (used Flutide inhaler and Nasonex nasal spray). About control subjects, one reported hypertension, gastrointestinal symptoms and arthritis (used sparkal, metoprolol, artox). Another control subject reported hypertension and diabetes (unknown medications). No information about the systemic condition was available for the last control subject.

### 3.2 Expression of MPO

**Figure 3:**
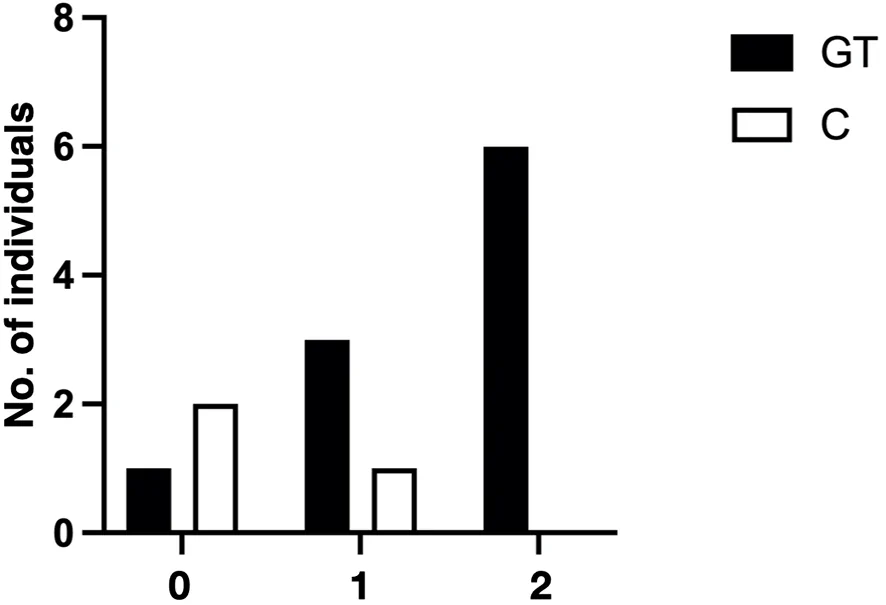
MPO expression among patients with GT and controls. MPO scoring was compared to positive controls i.e. tonsils as 0, negative; 1, weaker than tonsils; and 2, equivalent or stronger than tonsils.

The expression of MPO was assessed among ten patients with GT and three controls. The difference in the expression of MPO among GT patients and controls was statistically significant (*p* = 0.0266; **Figure 3**). Most of the patients with GT (6/10) had MPO score 2. In contrast, 2/3 control subjects were negative for MPO (score 0).

### 3.3 Expression of EGF and EGFR

**Figure 4:**
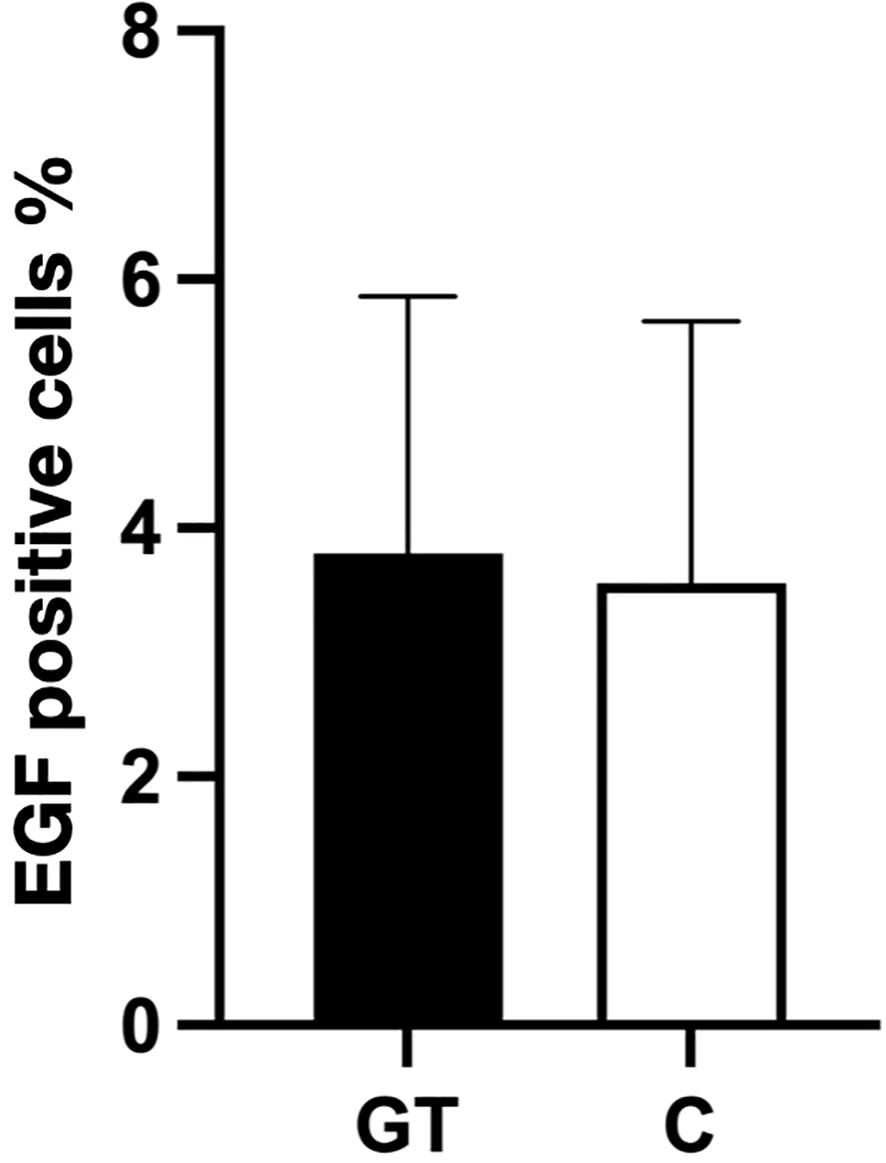
Expression of EGF positive cells among patients with GT and controls. Values shown are means ± SEM.

**Figure 5:**
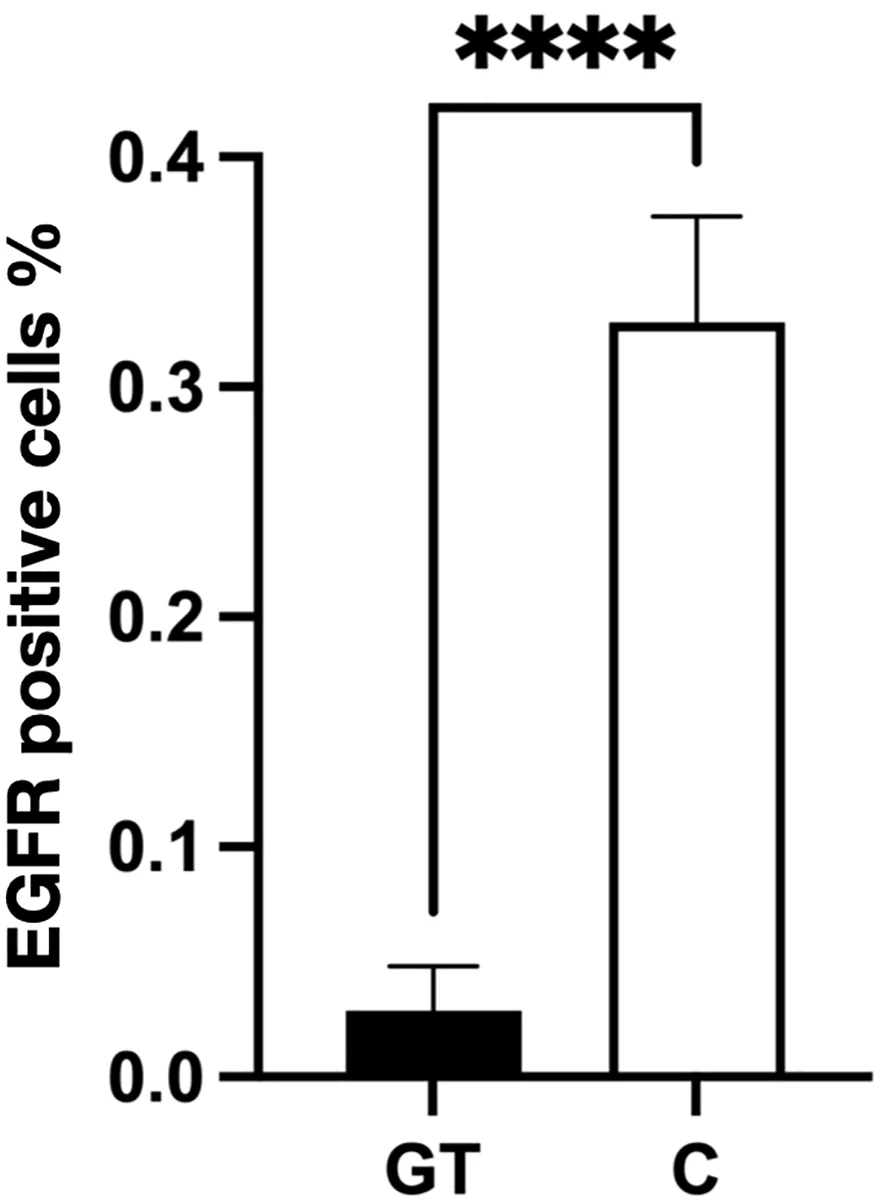
Expression of EGFR positive cells among patients with GT and controls. Values shown are means ± SEM. ****p < 0.0001.

The mean percentage of EGF positive cells were slightly higher in the tissue samples of tongue among patients with GT (n=8, 3.8% ± 2.1%) *vs.* controls (n=3, 3.5% ± 2.1%), however, without a significant difference between the two groups (*p* = 0.9; **Figure 4**). In contrast, the mean percentage of EGFR positive cells were lower in the tissue samples for patients with GT (n=8, 0.02% ± 0.01%) vs. controls (n=3, 0.3% ± 0.04%) representing a statistically significant difference (*p* < 0.0001; **Figure 5**). For both EGF and EGFR, 8 of 10 samples were analysed. The remaining two samples could not be analysed due to insufficient material.

## 4 Discussion

In the present study, we found significantly increased MPO expression and reduced EGFR activity in patients with GT compared with controls, whereas EGF expression did not differ between the groups. These findings suggest that GT is characterized by enhanced neutrophil-associated inflammation together with impaired activation of epithelial regenerative pathways.

The increased MPO expression observed in GT is likely explained by the neutrophilic infiltration previously described in this condition (12, 13), as MPO is predominantly stored in the azurophilic granules of neutrophils (6). This interpretation is further supported by our previous finding of elevated salivary IL-8 levels in GT (5), as IL-8 is a potent neutrophil chemoattractant (14). Persistent inflammation may therefore promote continuous recruitment of neutrophils to the affected tongue mucosa. Besides their antimicrobial functions, neutrophils contribute to restoration of epithelial barrier integrity and maintenance of tissue homeostasis during inflammation resolution (15). Although their precise role in GT remains unclear, neutrophils are recognized as key regulators of oral mucosal homeostasis and the oral ecosystem (16, 17).

MPO is a major component of the innate immune response owing to its potent antimicrobial activity (18). It should be noted that saliva contains several other peroxidase systems and antimicrobial proteins; therefore, MPO represents only one of the components of this antimicrobial network (19). Interestingly, alterations in the tongue microbiota have been reported in GT (20), suggesting that microbial dysbiosis may contribute to disease pathogenesis. Microorganisms colonizing the compromised tongue epithelium may stimulate cytokine production (21), leading to further neutrophil recruitment (22). Consequently, the increased MPO expression observed in the present study may reflect both enhanced neutrophil infiltration and activation of antimicrobial defence mechanisms.

Only a limited number of studies have investigated MPO in oral mucosal diseases. In OLP, MPO polymorphisms have been associated with disease susceptibility, suggesting that MPO-mediated oxidative activity may contribute to chronic inflammatory conditions characterized by persistent neutrophil infiltration (23). In contrast, no differences in salivary MPO levels were reported in patients with orofacial granulomatosis, with or without Crohn’s disease (24). MPO has been more extensively investigated in periodontal diseases and peri-implantitis, where elevated levels are considered to reflect ongoing inflammation and antimicrobial host defence (25, 26). The increased MPO expression observed in GT may therefore represent a similar neutrophil-driven inflammatory response.

The biological functions of EGF have been evaluated in experimental studies, which have demonstrated the importance of EGF in the maintenance of papillary morphology (11), wound healing (27), and the chemotaxis of vascular endothelial cells during tissue repair (28). EGFR signalling cascade is a key regulator in cell proliferation, differentiation, division, survival, as well as cancer development (10). Although EGF expression did not differ between groups, activated EGFR expression was significantly reduced in GT. Because ligand binding and receptor phosphorylation are required for downstream EGFR signalling (29), our findings suggest that impaired receptor activation rather than altered ligand availability may underlie defective epithelial regeneration in GT. This interpretation may also explain our previous observation of fluctuating salivary EGF concentrations (5). In addition, chronic inflammation in GT may further suppress EGFR signalling through MPO derived oxidants which can modify membrane proteins, including EGFR, thereby impairing receptor activation (30). It is conceivable that altered EGFR activation results in unstable epithelial signalling, thereby compromising regeneration of filiform papillae and contributing to the characteristic papillary atrophy observed in GT.

In contrast to our results of a decreased EGFR expression among GT patients, most of the abnormalities of an increased EGFR signalling or gene amplification were found to be associated with tumorigenesis and tumour progression (31). The expression of EGFR was studied extensively in oral squamous cell carcinoma (OSCC) and in oral potentially malignant disorders (31, 32). In OLP, controversial findings were reported about the expression of EGF and EGFR (33–35), which may correlate with the clinical type of OLP (36). In oral leukoplakia, an increased expression of EGF and EGFR was found to be associated with dysplastic lesions and with lesions at high-risk sites for malignant transformation (37–39). Moreover, genomic alteration of EGFR in oral leukoplakia was one of OSCC drivers that may drive the malignant progression (40). In similarities with oral leukoplakia, the gene expression of EGFR was found to be upregulated in oral submucous fibrosis (41, 42). Recently, there is a growing interest into the implications of EGF/EGFR as a target therapy in several diseases. In the field of oral mucosal lesions, the use of EGFR inhibitor as a target therapy was investigated in pemphigus vulgaris in murine models and in cultured keratinocytes in vitro model showing promising results (43–45).

The present study has several limitations. First, the number of tissue samples was relatively small, reflecting the fact that GT is a benign condition for which biopsies are rarely indicated. Consequently, irritational fibromas were used as control tissue. Although these lesions represent reactive rather than neoplastic changes and are not known to exhibit increased EGFR activity (46, 47), they are not equivalent to healthy tongue mucosa. Ethical considerations preclude biopsy sampling of healthy tongue tissue in asymptomatic individuals, limiting the availability of ideal controls. Future studies including larger cohorts and, where feasible, healthy tongue specimens would strengthen the evidence regarding the role of MPO and EGFR signalling in GT.

## 5 Conclusions

In conclusion, GT appears to be characterized by enhanced neutrophil-associated inflammation together with reduced EGFR activation, suggesting an imbalance between inflammatory responses and epithelial regeneration. Whether impaired EGFR signalling represents a primary pathogenic mechanism or a secondary consequence of chronic inflammation remains to be determined. These findings provide new insights into the biological mechanisms underlying GT and warrant further investigation in larger studies.

## Data Availability

The raw data supporting the conclusions of this article will be made available by the authors, without undue reservation.
